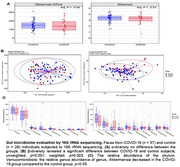# Increased Myeloid Progenitor Inhibitory Factor 1 and Dysbiosis in Mild COVID‐19 Patients With No Change in Alzheimer's Disease Markers

**DOI:** 10.1002/alz.091825

**Published:** 2025-01-09

**Authors:** Vijayasree V Giridharan, Celso Catumbela, Bruno Kluwe‐Schiavon, Camila O. Arent, Laura A. Borba, Margarete D. Bagatini, Zuleide M Ignácio, Lucas C Pedro, Luciane B Ceretta, João Quevedo, Rodrigo Morales, Gislaine Z Reus, Tatiana Barichello

**Affiliations:** ^1^ The University of Texas Health Science Center at Houston, Houston, TX USA; ^2^ University of Southern Santa Catarina, Criciuma Brazil; ^3^ Universidade Federal da Fronteira Sul, Chapeco Brazil; ^4^ University of Southern Santa Catarina (UNESC), Criciuma, SC Brazil; ^5^ The University of Texas Health Science Center at Houston (UTHealth), Houston, TX USA; ^6^ University of Southern Santa Catarina (UNESC), Criciúma Brazil; ^7^ Universidade do Extremo Sul Catarinense, Criciuma, SC Brazil

## Abstract

**Background:**

The persistent neurological symptoms seen in long COVID survivors are attributed to immune system dysfunctions and changes in the microbiome induced by SARS‐CoV‐2 infection. In addition to the initial respiratory manifestations, a significant portion of COVID‐19 patients present with neurodegenerative symptoms. Our hypothesis suggests that disruptions in inflammatory signals and alterations in the gut microbiome post‐COVID‐19 play pivotal roles in the development of neurodegenerative complications among individuals experiencing prolonged effects of the disease.

**Method:**

In our cross‐sectional study, we enrolled individuals with post‐COVID‐19 (n = 26) and age‐ and sex‐matched healthy controls (n = 57). Our objective was to conduct a comprehensive examination of neuropsychiatric symptoms, lipid profiles, oxidative stress markers, inflammatory signals, and Alzheimer's disease (AD) markers in plasma samples and gut microbiome status using feces samples from post‐COVID patients. Depressive and anxiety symptoms were evaluated using the Hamilton Rating Scale, while cognitive performance was assessed with standardized measures. The intricate details of the gut microbiome status were revealed through 16S rRNA sequencing.

**Result:**

The investigation uncovered no statistically significant differences in anxiety or cognitive status however the depression was elevated in post‐COVID‐19 as compared to healthy subjects. Further, AD markers (Aβ42, Aβ40, t‐tau, and p‐tau) and oxidative stress markers displayed no discernible distinctions between the two groups. A noteworthy discovery was the significant increase in CCL23, the macrophage inhibitory protein‐3, among COVID‐19 patients. CCL23, known for its role in inflammation and host defense, has recently been linked to neuroinflammation in the early stages of AD. Furthermore, apoptosis markers Caspase‐3 and ‐8 were significantly elevated in COVID‐19 patients. While α‐diversity in the gut microbiome showed no significant differences, β‐diversity indicated a notable distinction between the control and post‐COVID‐19 groups. Furthermore, post‐COVID‐19 individuals displayed a decreased abundance of the phylum Verrucomicrobia and genus Akkermansia, a short‐chain fatty acid producer, and microbial group significantly associated with intestinal barrier permeability and cognitive improvement.

**Conclusion:**

While longitudinal studies are essential for a comprehensive understanding of the behavioral trajectory of COVID‐19 individuals, the current results indicate that CCL23 levels and changes in microbiome status may function as early indicators of post‐COVID neurological and neuropsychiatric outcomes.